# Drugs Repurposing of Molecules Modulating Human Delta Globin Gene Expression via a Model of Transgenic Foetal Liver Cells: Implications for Beta-Hemoglobinopathy Therapeutics

**DOI:** 10.3390/biom15040565

**Published:** 2025-04-11

**Authors:** Michela Simbula, Maria Francesca Manchinu, Stefania Olla, Michela Congiu, Simona Vaccargiu, Cristian Antonio Caria, Daniela Poddie, Maria Serafina Ristaldi

**Affiliations:** Istituto Di Ricerca Genetica e Biomedica del Consiglio Nazionale Delle Ricerche (IRGB-CNR), 09042 Monserrato, Italy; michela.simbula@cnr.it (M.S.); mariafrancesca.manchinu@cnr.it (M.F.M.); stefania.olla@cnr.it (S.O.); michelacongiu@cnr.it (M.C.); simona.vaccargiu@cnr.it (S.V.); cristian.caria@irgb.cnr.it (C.A.C.); daniela.poddie@cnr.it (D.P.)

**Keywords:** beta-thalassemia, sickle cell disease, delta globin gene, drug assay

## Abstract

Beta-hemoglobinopathies such as beta-thalassemia and sickle cell disease are severe genetic blood disorders affecting the beta globin chain of haemoglobin A (α2β2). Activation of delta globin, the non-alpha globin of HbA2 (α2δ2), could represent a possible approach to improve the clinical severity of these pathologies. Notably, the therapeutic potential of delta globin has been demonstrated in previous studies using a mouse model of beta-thalassemia and sickle cell disease. The present study evaluated *delta globin* gene activation by small molecules in erythroid cells isolated from transgenic murine foetal liver. A screening of 119 molecules, selected for their potential in drug repurposing, was performed without prior selection based on specific pathways of interest. Three candidates—Nexturastat, Stattic and Palbociclib—were found to have high efficacy on *delta globin* expression. Palbociclib also proved effective in increasing *gamma globin* expression. All of these compounds have pharmacokinetic profiles that are beneficial for clinical application, providing potential inducer agents of HbA2 that could have therapeutic effects in the treatment of beta-hemoglobinopathies.

## 1. Introduction

Beta-hemoglobinopathies (β-hem) are the most common monogenic diseases in the world, affecting the health of millions of people worldwide [1,2]. Among them, sickle cell disease (SCD) and beta-thalassemia (β-thal) are the most prevalent, having the greatest public health impacts in economic terms [3].

SCD results from a single nucleotide substitution (SNS rs334) in the *beta globin* gene (HBB) that causes the formation of haemoglobin (i.e., HbS), which confers the characteristic sickle shape in erythrocytes, whereas beta-thalassemias can be caused by several mutations (single nucleotide substitution, deletions, insertions, etc.) in the *HBB* gene [4].

β-hem are distributed in high frequencies throughout many countries of the world—particularly in the Mediterranean region, South and Central America, Saudi Arabia, sub-Saharan Africa, and the Indian subcontinent. Notably, increasing β-hem incidence rates and their current geographic distribution are leading to severe dissemination. Current treatment options for β-hem include blood transfusions, iron chelation, foetal haemoglobin (HbF) induction and bone marrow transplantation. Although bone marrow transplantation remains the only definitive cure, its use is limited by donor availability and associated risks. Gene therapy and gene-editing techniques remain largely experimental, and alternative therapeutic approaches are urgently needed [5].

Numerous studies have focused on activating HbF (α2γ2), since it has been established that increasing HbF production can alleviate the clinical severity of these diseases [5]. Current approaches are primarily aimed at identifying pharmacological agents and novel strategies for inducing HbF expression.

Previous in vitro and in vivo studies by our group [6,7] and others [8,9] have shown that haemoglobin A2 (HbA2, α2δ2) also has therapeutic potential. Although HbA2 is typically expressed at low levels, it is fully functional and improves SCD and β-thal features in animal models, thereby validating the *delta globin* gene as a potential therapeutic target [6,7]. Therefore, activating the human *delta globin* gene may represent a viable therapeutic strategy for treating β-thal and SCD. Additionally, prior work from our laboratory has also established the proof of principle that the *delta globin* gene can be induced following treatment with drugs, namely, interferon beta [10].

In the context of identifying new treatments, drug repurposing offers a promising avenue for accelerating therapeutic development. In this work, we have conducted a screening of existing approved drugs, or those in clinical trials, to determine whether they can activate or modulate HbA2 expression, which could be beneficial in managing β-hem. Drug repurposing leverages the safety profiles of established medications, significantly reducing the time and cost associated with bringing new treatments to the clinic setting.

The use of animal testing has been essential for advancing our understanding of β-hem, developing treatments and testing new therapies. However, this practice is not without ethical and practical problems. For these reasons, we decided to establish an alternative ex vivo model as a viable option to study and test new therapeutic strategies. In the present study, we developed a two-stage screening cellular model for identifying potentially therapeutic active small molecules, circumscribing the number of molecules to be evaluated in vivo.

To verify whether *delta globin* gene expression can be increased by molecules, we created two mouse foetal liver cell lines derived from experimentally validated transgenic mice. First, we developed a foetal liver cell line derived from a dual-reporter transgenic mouse [6], which allowed us to complete the rapid screening of several molecules and select a few that were effective. The selected molecules were then screened at the mRNA level on a second foetal liver cell line derived from a transgenic humanised mouse model (Ln72) bearing the full *beta globin* gene cluster [11,12]. Both cellular models faithfully reproduce the globin gene expression pattern of the in vivo models from which they are derived.

The creation of cell lines from specific tissues of interest—derived from transgenic mouse models that are already thoroughly studied and experimentally validated—offers a compromise between the need for research and animal welfare. By balancing the need for robust scientific data with animal welfare, this approach provides reliable data while supporting the principles of the 3Rs (replacement, reduction, and refinement) [13].

The results of this study allowed us to select three molecules to be evaluated for repurposing in β-hem therapy in preclinical disease mouse models. We also created cell lines useful for investigating and developing new therapeutic strategies for these diseases. The repositioning of molecules, which may increase *delta globin* gene expression, would result in possible applications for patients with the improvement of their quality of life and life expectancy.

## 2. Materials and Methods

### 2.1. Selection Compounds for Screening

For compound screening, two distinct libraries of compounds were selected. The first library consists of 101 compounds from the L1900 library of epigenetic modulators (Table S1) (www.selleckchem.com/screening/epigenetics-compound-library.html, accesssed on 7 March 2023). The original L1900 library contains 914 compounds, which we considered too large for a primary analysis. Therefore, we narrowed the selection to 101 compounds. The arbitrary selection of compounds was based on two main criteria: (1) the diversity of mechanisms of action by excluding compounds with the same target; (2) the chemical diversity by excluding molecules with a similar chemical class. This process allowed us to maintain a broad range of targets and significant chemical variability, optimising the efficiency of the initial screening. The second library comprises 18 compounds targeting cell cycle modulation (Table S2). For this library, compounds were chosen focusing on well-characterised targets connected to cell cycle regulation. Only those compounds with a known association with the cell cycle and that were commercially available were selected for screening.

### 2.2. In-Silico Prediction of Chemical-Physical Properties of Drugs

The Absorption, Distribution, Metabolism, Excretion and Toxicity (ADME-Tox) profile of the investigated compounds was predicted through the Schrodinger QikProp tool (Schrodinger, Release 2022-3). Some of the most important properties used to check whether compounds had suitable properties include the partition coefficient (QP log P octanol/water), human oral absorption rate, activity in Central Nervous System (CNS), MDCK (Madin–Darby canine kidney cell used for CNS absorption and predict CNS access) and Caco-2 apparent permeability (intestinal absorption), and “druggability” indication (Lipinski Rule of Five violations and Jorgensen Rule of 3 Violations).

### 2.3. Mice

The transgenic mice carrying human mutated delta promoter, CACCCδ-LCR, and WT promoter δ-LCR were generated as described [6]. P53 null and Ln72 transgenic mice were kindly provided by Dr. Frank Grosveld’s laboratory [12]. The genotypes for delta transgenic mice, P53 (WT, heterozygous and KO) and Ln72 were determined by polymerase chain reaction using the primers listed in Table S3. All procedures conducted on the animals were in accordance with the rules and regulations set by the Ethical Committee (OPBA) of University of Cagliari (approval number: 781/2021_EXT.1), 18 October 2023.

### 2.4. Cell Lines Generation and Culture

Cell lines were generated from HS2δFLβRL, HS2CACCCδFLβRL and Ln72 transgenic mouse lines. The HS2δFLβRL transgenic mouse line contains the promoters of the *delta* and *beta globin* genes driving the expression of the reporter genes Firefly and Renilla luciferase, respectively (Figure S1). Using a dual Firefly/Renilla luciferase reporter construct for the human *delta/beta globin* gene promoter allowed us to study their relative expression variations. The HS2CACCCδFLβRL transgenic line was used as a positive control for delta globin gene promoter activation in our assay since this promoter has been mutagenised to contain the CACCC sequence. The CACCC box is a binding site for the activating transcription factor KLF1 [14]. The Ln72 transgenic mouse line carries the complete human *beta globin* gene cluster [12].

The culturing of primary foetal liver cells and the generation of transgenic cell lines were carried out as described [11]. Briefly, foetal livers of day 14.5 to mouse embryos (p53−/− HS2δFLβRL, p53−/− HS2δCACCCFLβRL and p53−/− Ln72) were resuspended in 1mL serum-free stem cell expansion medium (StemPro-34^TM^; Life Technologies Europe BV). Cells were passed through a 70 mm Nylon cell strainer (Euroclone-Italy), washed and seeded at 4 × 10^6^ cells/mL into Stem-Pro-34^TM^ (Life Technologies Europe BV) medium supplemented with human erythropoietin (Eprex 4000 UI/4 mL, 2 U/mL), murine recombinant stem cell factor (SCF, Sigma aldrich, USA, 100 ng/mL) and dexamethasone (Dex, Sigma-aldrich, USA, 10^−6^ M); cell density was maintained between 3 and 7 × 10^6^ cells/mL by daily dilution with fresh medium containing 2× factors [13]. To induce differentiation, cells were washed twice in PBS and seeded at 3 × 10^6^ cells/mL in Stem-Pro-34^TM^ medium supplemented with human erythropoietin (Eprex 4000 UI/4 mL, 10 U/mL) and transferrin (Sigma Aldrich USA 500 μg/mL).

### 2.5. Luciferase Assay

The Dual Luciferase Reporter Gene Assay (Promega USA) was used to determine the activity of a delta and beta promoter according to the manufacturer’s instructions.

Reporter assays were carried out 72 h after compound treatment; we tested the library of 101 epigenetic modulators and the 18 cell cycle modulators compounds at 10 μM. Active molecules at this concentration have been tested at 1 μM and 0.1 μM. The relative luciferase activity was measured using the Dual-Luciferase Reporter Assay System (Promega USA) and a Synergy 2 Plate Reader (BioTek- USA). Firefly luciferase activity was normalised to Renilla luciferase activity. All the experiments were independently repeated six times for statistical validation. Luciferase activity obtained from HS2δFLβRL and HS2δCACCCFLβRL cells line was used as a negative and positive control, respectively.

### 2.6. Real-Time Quantitative PCR (RT-qPCR)

RT-qPCR was carried out 72 h after compound treatment at 10 μM and 30 μM. Total RNA was isolated using TRIzol reagent. The cDNA was made from total RNA by performing DNAse treatment (Invitrogen Europe BV) and then using Superscript III reverse transcriptase (Invitrogen Europe BV).

RT-qPCR was performed to measure the beta, gamma and *delta globin* gene mRNA expression, and samples were normalised against murine *alpha globin* gene levels. The gene’s expression was detected by Sybr Green chemistry (Invitrogen Europe BV). All primers used for the RT-qPCRs are listed in Table S3.

Real-time PCR was carried out using an ABI PRISM 7900 thermocycler (Applied Biosystems, Foster City, CA, USA). The reactions were performed on at least three different samples in triplicate. The analysis of RT-qPCR data was performed using the ΔΔCT method.

### 2.7. Statistics

All statistical differences were calculated using the unpaired Student’s *t*-test or One-way ANOVA with Bonferroni correction, using Prism (GraphPad, USA) version 10.4.

## 3. Results

### 3.1. Creation of Foetal Liver Cell Lines for Drug and Small Molecules Screening

To identify drugs capable of enhancing *delta globin* gene expression, we developed two ex vivo models using murine foetal liver cells isolated at 14.5 days post-coitum (dpc). These cells were derived from transgenic mouse models crossed in homozygosity with a p53 knockout background, as previously described [11]. The generated cells represent a physiological analogue of an erythroid progenitor in terms of transcriptional regulation and proper erythroid differentiation [15]. For the first cell model, we used transgenic mouse lines containing the constructs HS2δFLβRL and HS2CACCCδFLβRL, which were created by our laboratory and previously described [6] (Figure S1).

**Figure 1:**
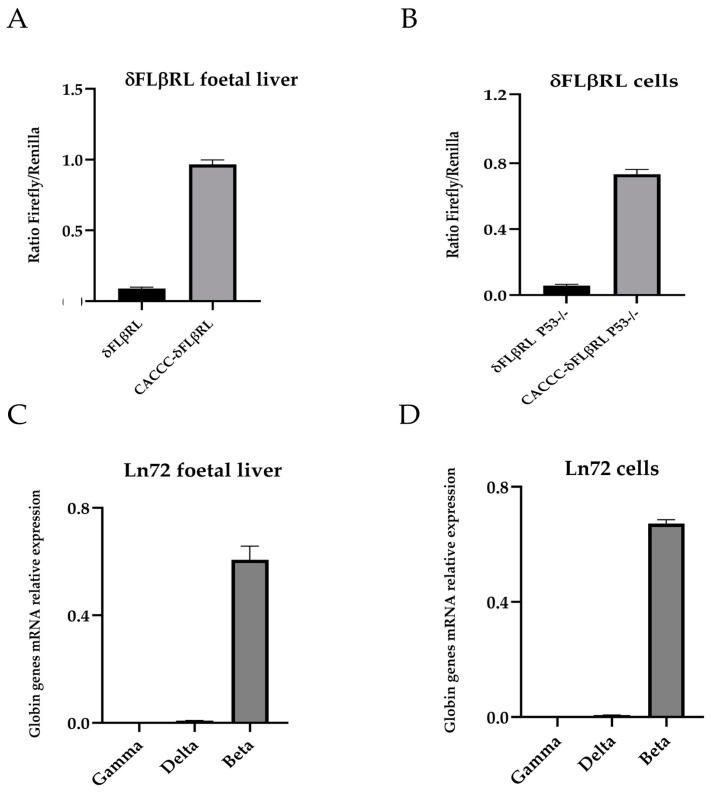
Ratio Firefly/Renilla and *gamma, delta* and *beta* globin genes expression analysis. (**A**) Ratio Firefly/Renilla in foetal liver cells of the two transgenic mouse models δFLβRL (HS2δFLβRL) and CACCCδFLβRL (HS2CACCCδFLβRL) (14.5 dpc). (**B**) Ratio Firefly/Renilla in the ex vivo δFLβRL cells. (**C**) Relative mRNA expression levels of *gamma, delta* and *beta* globin genes in Ln72 foetal liver (14.5 dpc). (**D**) Relative mRNA expression levels of *gamma, delta* and *beta* globin genes in Ln72 ex vivo cells (14.5 dpc). Levels were quantified by RT-qPCR, and results are expressed as the value relative to the alpha mouse, (n = 6 for each group).

To determine whether these cells (hereafter referred to as δFLβRL cells) serve as a physiological analogue of erythroid progenitors with respect to accurate transcriptional regulation—an essential factor for globin gene expression studies and drug assays in the context of drug discovery and repositioning—we analysed the Firefly/Renilla ratios in the foetal liver cells of the two original transgenic mouse models and compared them to that of the δFLβRL cells. As shown in Figure 1A,B, our data confirm that the Firefly/Renilla ratio of δFLβRL cells is similar to that of the original transgenic mice HS2δFLβRL and HS2CACCCδFLβRL foetal liver at the same developmental stage (14.5 dpc). These cells were then used to perform a primary screening of small molecules by reporter assay, as described later.

To further validate the results via a secondary mRNA-based screening, using the active molecules selected by the primary screening, we developed a second ex vivo foetal mouse cell model (hereafter referred to as Ln72 cells) derived by intercrossing a humanised mouse model (Ln72) carrying the complete *beta globin* gene cluster and a p53 knockout mouse. The mRNA expression levels of gamma, delta and beta globin genes in Ln72 cells were quantified by RT-qPCR and compared to that obtained from the original Ln72 transgenic mouse model. As shown in Figure 1C,D, the expression levels of *gamma, delta* and *beta* globin genes in Ln72 cells are similar to that of the humanised Ln72 mouse foetal liver cells at the same developmental stage (14.5 dpc).

These results validated δFLβRL and Ln72 cells as a physiological analogue of the in vivo model (from which they are derived) in terms of the consistency of transcriptional regulation, thereby making them suitable for evaluating the potential of active molecules in increasing *delta* globin gene expression.

### 3.2. Screening of Drugs by Luciferase Assay via δFLβRL Foetal Liver Cells

To perform a screening for molecules able to increase *delta globin* gene expression, we used δFLβRL cells, obtained as described above (Figure S1).

**Figure 2:**
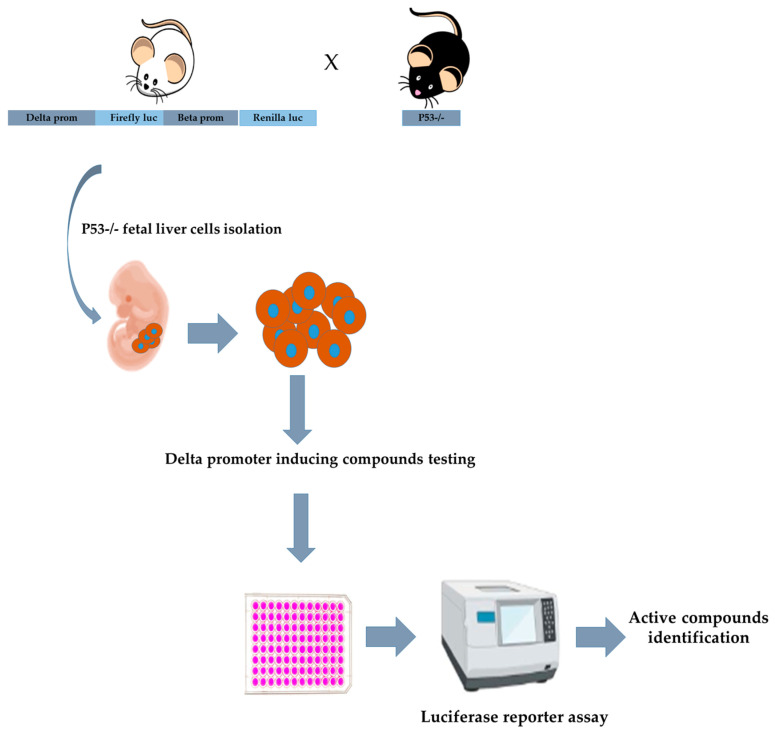
Schematic workflow of the high-throughput screening assay (HTS).

Figure 2 presents the workflow for screening small molecules using a luciferase assay.

To screen for potential modulators, we considered two classes of compounds: epigenetic and cell cycle modulators. Epigenetic modulators were chosen because their role in regulating *globin* gene expression is well-established [16]. Cell cycle modulators were chosen because previously published data from our research group and others indicate that the expression levels of delta and gamma globin are tightly linked to the cell cycle [17,18].

We tested a selected custom library of 101 compounds known as epigenetic modulators (see Section 2 and Table S1) and 18 compounds selected as cell cycle modulators (see Section 2 and Table S2).

The combination of the two selected libraries covered a wide range of targets and chemical diversity, optimising the chances of identifying promising compounds in our study.

**Figure 3:**
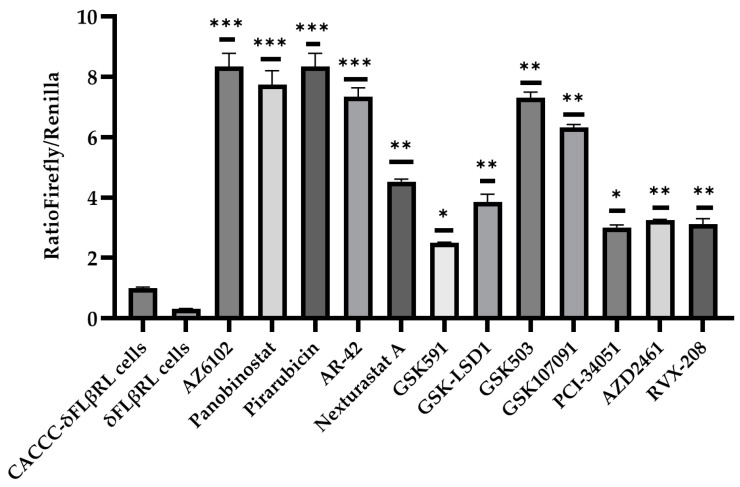
Ratio Firefly/Renilla epigenetic modulators. The histogram shows the ratio Firefly/Renilla after compound treatment in foetal 14.5 liver cells. Cells containing the CACCC promoter were used as a positive control, while cells carrying the WT delta promoter were used as a negative control and were treated with DMSO only. Levels of significance calculated by One-way ANOVA with Bonferroni correction are indicated (* *p* < 0.05, ** *p* < 0.01, *** *p* < 0.001).

δFLβRL cells were induced to differentiate by adding erythropoietin and transferrin (see Section 2) and then treated with each compound at a single 10 μM concentration in a 96-well plate for a pivotal study. After 72 h, cells were collected and analysed. We performed three independent experiments testing the library of 101 epigenetic modulators. Overall, 12 of the tested modulators showed the significantly increased activity of the *delta globin* gene promoter when compared to the beta (see Figure 3). We also performed the same assay for 18 selected cell cycle modulators.

**Figure 4:**
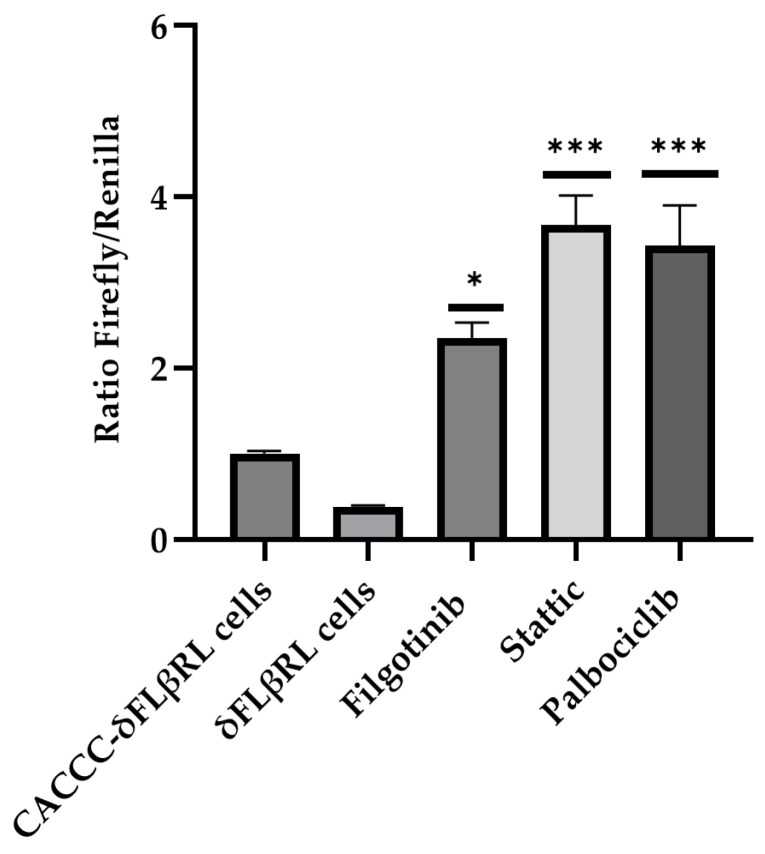
Ratio Firefly/Renilla cell cycle modulators. The histogram shows the ratio Firefly/Renilla after compound treatment in foetal 14.5 liver cells. Cells containing the CACCC promoter were used as a positive control, while cells carrying the WT delta promoter were used as a negative control and were treated with DMSO only. Levels of significance calculated by One-way ANOVA with Bonferroni correction are indicated (* *p* < 0.05, *** *p* < 0.001).

As shown in Figure 4, three compounds demonstrated an increased activity when compared to the control. The 15 selected active molecules, comprising 12 epigenetic modulators and 3 cell cycle modulators, were subsequently analysed in more detail through a dose-response evaluation, using three different concentrations (10, 1 and 0.1 µM). Our data indicate that, at 1 and 0.1 µM, the compounds did not display significant activity.

By testing numerous compounds to identify hit compounds for therapeutic purposes, this initial screening enabled us to identify 15 molecules capable of activating the *delta globin gene* promoter. To further confirm our findings, the 15 active compounds were also tested on Ln72 foetal liver cells to the mRNA level, providing additional validation of our approach.

### 3.3. Validation of Active Drugs via Ln72 Foetal Liver Cells

**Figure 5:**
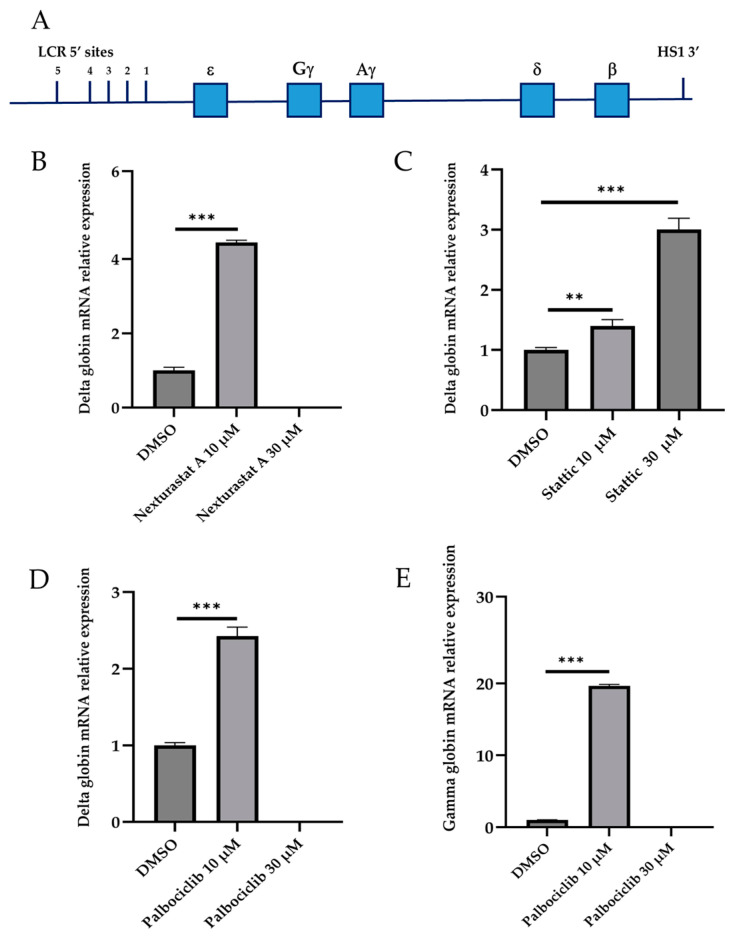
Gamma, delta and beta mRNA levels in Ln72 ex vivo cell line. (**A**) Schematic representation of the humanised mouse model (Ln72) bearing the full *beta globin* gene cluster used in the present study. (**B**) Relative expression levels of *delta globin* gene in Ln72 cell line (14.5 dpc) treated with Nexturastat. (**C**) Delta expression levels in Ln72 cell line (14.5 dpc) following Stattic treatment. (**D**,**E**) Analysis of delta and gamma expression in the Ln72 cell line (14.5 dpc) under Palbociclib treatment. Levels were quantified by RT-qPCR, and results are expressed as the value relative to the alpha mouse. Levels of significance calculated by *t*-test are indicated ( ** *p* < 0.01, *** *p* < 0.001); (n = 4 for each group).

The screening of molecules using a reporter system allowed us to select 15 active molecules, 12 epigenetic modulators and 3 cell cycle modulators (Figure 3 and Figure 4). To validate the activity of these 15 small molecules at the transcriptional level and in the context of the normal human *beta globin* gene cluster, we established a foetal liver (14.5 dpc) derived cell line from a humanised mouse model (Ln72) bearing the full beta globin gene cluster (Figure 5A) by crossing it with the P53−/− mouse line, as previously described.

We measured the *delta, gamma* and *beta* globin genes’ mRNA expression levels using RT-qPCR in cells treated with the 15 compounds shown to be active in the preliminary screening with the dual-reporter assay. Each compound was tested at concentrations of 10 and 30 μM. The higher dose of 30 μM was used to evaluate whether the efficacy of the molecules exhibited a dose-dependent increase. Twelve compounds showed no activity on delta, gamma and beta globin gene expression, while the compounds Nexturastat (epigenetic modulator), Stattic, and Palbociclib (cell cycle modulators) showed significant activity.

Our results demonstrate that, in Ln72 cells, the *delta globin* gene expression level significantly increased following treatment with 10 μM Nexturastat, achieving a 4.44 ± 0.06-fold increase (*p* = 2.57 × 10^−5^; Figure 5B). However, cytotoxic effects were observed at a concentration of 30μM. No activation of the *gamma globin* gene was detected under Nexturastat treatment.

Stattic treatment resulted in a significant increase in *delta globin* gene expression, with a 1.39 ± 0.10-fold change at 10μM (*p* = 0.017) and a marked increase to 3.00 ± 0.18-fold at 30 μM (*p* = 6.82 × 10^−5^; Figure 5C). No activation of the *gamma globin* gene was detected under Stattic treatment.

As illustrated in Figure 5D, treatment with Palbociclib significantly increased *delta globin* gene expression at 10 μM, with a fold change of 2.42 ± 0.11 (*p* = 0.0005) when compared to the control. Furthermore, Palbociclib induced a remarkable increase in *gamma globin* gene expression at the same concentration, reaching a 19.66 ± 0.19-fold change (*p* = 2.07 × 10^−7^; Figure 5E). Similar to Nexturastat, cytotoxic effects were noted at 30 μM. None of the tested compounds significantly affected beta globin mRNA levels.

### 3.4. In Silico Prediction of Chemical–Physical Properties of Drugs

The absorption, distribution, metabolism, excretion and toxicity (ADME-Tox) properties of the 12 epigenetic modulators and of three compounds that affect the cell cycle were predicted using QikProp. Detailed information is provided in the Supplementary Materials (Tables S4 and S5). In Table 1, we specifically highlight key pharmacokinetic properties relevant to our study of the three active compounds in all assays. We examined several critical pharmacokinetic parameters, including lipophilicity, measured by the n-octanol–water partition coefficient (LogP), cellular permeability, evaluated through Caco-2 permeability for intestinal absorption, and the Madin–Darby canine kidney (MDCK) permeability for Central Nervous System (CNS) absorption.

Additionally, we assessed oral absorption within the gastrointestinal tract, the number of metabolites and the compounds’ activity in the CNS. We also evaluated druggability indicators, such as violations of Lipinski’s rule of five and Jorgensen’s rule. Furthermore, we analysed the number of property values or descriptors that fall outside the 95% confidence interval for similar values observed in known drugs (designated as #starts) and the count of reactive functional groups (designed as #rtvFG). All three compounds exhibited good pharmacokinetic properties and druglikeness (Table 1). However, the compound Stattic showed two descriptors outside the acceptable stats ranges, which are the ionisation potential (IP) and binding energy (EV) values. None of the three compounds showed activity in the CNS, which is a positive aspect since it reduces the likelihood of central side effects. Additionally, all three compounds demonstrated good oral absorption and decent cellular permeability.

## 4. Discussion

Previous in vitro and in vivo studies by our group [6,7], as well as by other groups [8,9], lead us to believe that the human *delta globin* gene activation could represent a valid treatment for β-hem as an alternative approach. Additionally, our prior research has demonstrated that the delta globin gene can be induced after treatment with specific molecules [10]. To date, however, interferon beta remains the only agent known to modulate *delta globin* gene expression [10]. Despite this, interferon beta is a non-selective drug associated with significant side effects and challenges in administration, thereby limiting its practicality and long-term use in clinical settings. These limitations underscore the need to develop molecules that can selectively and effectively activate *delta globin* gene expression.

This study aimed to repurpose existing drugs, specifically focusing on epigenetic and cell cycle modulators as activators of *delta globin* gene expression for potential therapeutic use in beta-thalassemia and SCD. For this study, we performed our screening on transgenic mouse-derived foetal liver cells. These mouse foetal liver cells represent a physiological analogue of an erythroid progenitor in terms of proper transcriptional regulation and erythroid differentiation [11].

The compounds identified in this screening, which include a selective HDAC6 inhibitor (Nexturastat), a STAT3 inhibitor (Stattic) and a CDK4/6 inhibitor (Palbociclib), have shown promising potential in inducing *delta globin* gene expression, offering a novel approach to address the globin imbalance and sickling characteristic of β-hem. Each compound appears to modulate *delta globin* gene transcription through distinct interactions with key regulatory pathways involved in cell growth and differentiation.

Nexturastat is a selective HDAC6 inhibitor that affects histone acetylation, a critical mechanism of epigenetic regulation. Non-selective HDAC inhibitors have previously been implicated in promoting HbF expression by altering the chromatin structure around *globin* genes, making them more accessible for transcription [19]. Some HDAC inhibitors, such as panobinostat, vorinostat and CT-101, have shown promising potential in inducing HbF expression in SCD and beta-thalassemia [5]. However, there is no evidence linking HDAC6 inhibitors—including Nexturastat—to the induction of HbF or *gamma globin* gene expression, and no effect was observed in the present study. Unlike non-selective HDAC inhibitors, Nexturastat primarily acts on cytoplasmic substrates rather than nuclear histones, thereby limiting its direct effect on chromatin remodelling and transcriptional regulation [20,21].

In mouse foetal liver at 14.5 dpc, the human *delta globin* gene is in an open chromatin conformation since it is actively transcribed, whereas the gamma globin gene is in a closed conformation since it is not transcribed. Nexturastat may indirectly affect the chromatin structure at the delta globin gene locus, promoting a more open conformation and increasing the transcription of delta globin. However, it likely cannot sufficiently alter the gamma globin gene’s chromatin to promote its expression.

Stattic, a transcription factor involved in inflammation and cancer, is primarily known for inhibiting the activation and the phosphorylation of STAT3. In this study, Stattic showed promising results, with a dose-dependent activation of the *delta globin* gene achieving up to a 3-fold increase when compared to the control at a concentration of 30 μM.

Although STAT3 has been reported to act as a negative regulator of *gamma globin* gene expression [22,23], our assay did not detect any changes in *gamma globin* gene expression. Notably, the mechanism by which Stattic increases *delta globin* gene expression remains unclear. This could be due to a direct effect through interaction with delta globin regulatory regions, or indirect, mediated by the activation or modulation of other transcription factors. Interestingly, recent studies have suggested that STAT3 could also be implicated in the regulation of erythropoiesis [24]. Therefore, by inhibiting STAT3, Stattic may favour *delta globin* gene expression through mechanisms related to erythroid differentiation.

Palbociclib, a potent inhibitor of CDK4/6, primarily functions to halt cell cycle progression at the G1 phase. Progression through the cell cycle from G1/G0 to the S, G2 and M phases is initiated by these cyclin-dependent kinases. CDK4 and CDK6 form a complex with one of their activating subunits, which are the cyclins D1, D2 and D3 [25,26]. Our lab has recently shown that gamma and *delta globin* gene expression are increased in Cyclin D3 KO mice [18]. The inhibition of CDK4/6 by Palbociclib produces a reduction in the number of terminal cell divisions and increases in cell size in mice foetal liver cells and human erythroid cultures from adult bone marrow progenitors, similar to the Ccnd3 null phenotype [27]. Accordingly, our data show that the extent of activation of the *gamma* and *delta globin* genes upon Palbociclib treatment is similar to that observed in the Cyclin D3 KO mice [18]. Treatment with Palbociclib may result in the production of mature erythrocytes with elevated levels of gamma and delta globin due to reduced cell divisions, thereby accelerating the differentiation process and thus favouring the expression of the gamma and delta over the *beta globin* gene [8,28,29]. Notably, the combined effect of the two globins could make this drug especially attractive for the treatment of SCD since HbA2 has comparable antisickling power to HbF.

The preclinical development of new drugs necessitates the use of animal models. Animal experimental models have been instrumental in advancing biomedical research, particularly in preclinical studies, where they provide crucial insights into complex biological processes and predict treatment outcomes [30].

Mice have long been the animal of choice for studying haemoglobin switching and genetic lesions of the *beta globin* gene, resulting in β-hem [31]. Humanised mice that possess a copy of the human *beta globin* gene locus have been generated [32,33]. Also, several models of beta-thalassemia and SCD have been developed over the past 40 years [34,35] and have been used for studies on the pathophysiology of the disease and to validate potential new therapeutic strategies.

In the present study, we used an experimental model involving two consecutive cellular screenings. The first involved an assay based on the use of reporter genes, which was useful for screening a large number of molecules. The molecules selected using reporters were then further evaluated through a secondary screening via a cellular model that contains the full human *beta globin* gene cluster in its entirety (Ln72). This allowed us to more stringently select potential active molecules for subsequent in vivo preclinical evaluation, thus reducing the use of animal models.

Prior to in vivo preclinical evaluation, it could be beneficial to evaluate the selected molecules in human CD34+ haematopoietic stem and progenitor cells (HSPCs). However, we utilised foetal erythroid liver cells from transgenic mice, which have been extensively characterised and studied, closely mimicking human erythropoiesis and *globin* gene expression patterns [6,12]. Our data, as well as those published by others [11], demonstrate that these cells faithfully replicate both the erythroid differentiation pattern and globin expression pattern observed in vivo in the transgenic mouse model from which they derive, thus representing a valuable tool for molecule screening.

This screening workflow could be applied to large-scale small molecule evaluation for the discovery of novel therapeutics for β-hem as well as for the discovery of new targets and pathways.

One significant advantage of repurposing Nexturastat, Stattic and Palbociclib lies in their established pharmacokinetic profiles, which are well understood from their current therapeutic use. These compounds exhibit favourable pharmacokinetics, including acceptable bioavailability and metabolic stability, which are key factors for their efficacy and safety in a clinical context.

Notably, among the three compounds, Palbociclib is already an approved drug used as an anti-cancer therapy, providing a robust clinical foundation for its repurposing. Clinical trials have reported that treatment with palbociclib may have some effect on haematological values [36,37]. However, to our knowledge, there are currently no data on the effects of palbociclib on HbF and HbA2 levels. In contrast, Stattic and Nexturastat have been studied primarily in preclinical settings, demonstrating potential in modulating disease-relevant pathways. This distinction highlights the translational readiness of Palbociclib when compared to the other two compounds.

Moreover, an important finding of this study is that these compounds do not demonstrate significant CNS activity, which is particularly advantageous for treating peripheral diseases such as beta-thalassemia and SCD. While desirable in neurological disorders, CNS penetration could lead to unnecessary side effects when treating peripheral blood disorders. The peripheral restriction of these drugs enhances their safety profile, making them more suitable for long-term use in patients suffering from hemoglobinopathies.

## 5. Conclusions

In our previous work, we validated the human *delta globin* gene as a therapeutic target for β-hem. In this work, we completed a pilot study aimed at repositioning molecules active on its expression. We identified three active compounds. Of these, Palbociclib seems to be the most promising as it is active on both the gamma and delta globin genes and is already used as a drug in patients with other pathologies.

Currently, therapeutic strategies for β-hem have progressed beyond conventional transfusion and chelation therapies, incorporating gene therapy and hematopoietic stem cell transplantation, which offer potential curative solutions for select patients. Despite their promise, these advanced treatments pose challenges, such as limited accessibility and the necessity for prolonged follow-up care [38]. Therefore, as these innovative options remain inaccessible to the majority of patients, particularly those in resource-limited regions, there is a pressing need for drug therapies that are safe, effective and affordable.

## Figures and Tables

**Table 1:** In silico pharmacokinetic parameters for Stattic, Palbociclib and Nexturastat.

Principal Descriptor	Range 95% of Drugs	Stattic	Palbociclib	Nexturastat
**#starts**	0 to 5	2	0	0
**#rtvFG**	0 to 2	0	0	1
**Predicted CNS Activity**	a −2 (inactive) to +2 (active)	−2	0	−2
**Apparent MDCK Permeability (nm/s)**	(<25 po or, >500 great)	67.78	62.49	145
**Apparent Caco-2 Permeability (nm/s)**	(<25 po or, >500 great)	156.88	134.31	216.72
**QP logP for octanol/water**	(−2.0/6.5)	−0.19	2.13	2.285
**No. of Primary Metabolites**	(1.0/8.0)	1	3	2
**Human Oral Absorption**	1 low, 2 medium, 3 high	2	3	3
**% Human Oral Absorption in GI**	(<25% is poor)	65%	78%	82%
**Lipinski Rule of 5 Violations**	(maximum is 4)	0	0	0
**Jorgensen Rule of 3 Violations**	(maximum is 3)	0	0	0

#starts: Number of property or descriptor values that fall outside the 95% range of similar values for known drugs. #rtvFG: Number of reactive functional groups.

## Data Availability

Data are available from the corresponding author upon reasonable request.

## Supplementary Materials

The following supporting information can be downloaded at: https://www.mdpi.com/article/10.3390/biom15040565/s1, Table S1: Library of epigenetic modulators; Table S2: Library of cell cycle modulators; Table S3: List of Primers used in this paper; Table S4: In silico ADME studies of 12 epigenetic modulators; Table S5: In silico ADME studies of 3 cell cycle modulators. Figure S1: Schematic representation of the construct used in the present study.

## Abbreviations

The following abbreviations are used in this manuscript:

SCD	Sickle cell disease
HBB	Beta globin gene
3Rs	Replacement, reduction, and refinement
DPC	Days post-coitum
ADME-Tox	Absorption, distribution, metabolism, excretion and toxicity
MDCK	Madin-Darby canine kidney
CNS	Central Nervous System
IP	Ionisation potential
EV	Energy values
