# Supplementary material for: Drugs Repurposing of Molecules Modulating Human Delta Globin Gene Expression via a Model of Transgenic Foetal Liver Cells: Implications for Beta-Hemoglobinopathy Therapeutics
Source: Biomolecules. 2025 Apr 11;15(4):565. doi: 10.3390/biom15040565 (PMC12025224; doi:10.3390/biom15040565)
Supplement: Supplementary file 1 [file biomolecules-15-00565-s001.zip › SUPPLEMENTARY FIGURE S1 and TABLE S3.pdf]

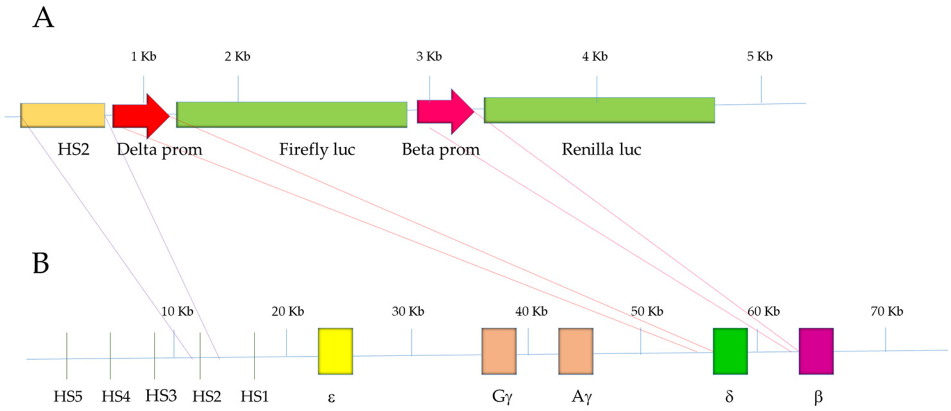

Figure S1. Schematic representation of the construct used in the present study. (A) Schematic representation of the HS2δFLβRL construct used for *ex vivo* reporter analysis. The construct is composed of a single HS2 enhancer, the delta globin gene promoter WT (HS2δFLβRL) or the CACCC containing delta globin gene promoter (HS2δCACCCFLβRL) driving Firefly luciferase and the Beta globin gene promoter driving Renilla luciferase. (B) Schematic representation of the human Beta cluster.

Table S3. List of Primers used in this paper.

GENE	SPECIES	APPLICATION	PRIMER SEQUENCE (5′ → 3′)
DELTA	Human	Genotyping	GCGGTGGGGAGATATGTAGA<br>GCCTTATGCAGTTGCTCTCC
P53	Mouse	Genotyping	TATACTCAGCCT<br>ACAGCGTGGTGGTACCTTAT<br>CATTCAGGACATAGCGTTGG
Ln72	Human	Genotyping	TAAGCCAGTGCCAGAAGAGC<br>TGATACCAACCTGCCCAGG
BETA	Human	RT-qPCR	TTGGACCCAGAGGTTCTTTGA<br>TCACTAAAGGCACCGAGCACT
GAMMA	Human	RT-qPCR	CTGAGTGAACTGCACTGTGACAAG<br>TCTTTGCCGAAATGGATTGC
DELTA	Human	RT-qPCR	TGAAACCCTGCTTATCTTAAACCAAT<br>TATGTCAGAAGAAAGTGTAAGCAACAG
ALPHA	Mouse	RT-qPCR	AGAGTCGAGTGGAAAGCACA<br>GAAGAGCAGACCCAGACGAT
